# Birth weight and diazoxide unresponsiveness strongly predict the likelihood of congenital hyperinsulinism due to a mutation in *ABCC8* or *KCNJ11*

**DOI:** 10.1530/EJE-21-0476

**Published:** 2021-10-30

**Authors:** Thomas I. Hewat, Daphne Yau, Joseph C. S. Jerome, Thomas W. Laver, Jayne A. L. Houghton, Beverley M. Shields, Sarah E. Flanagan, Kashyap A. Patel

**Affiliations:** 1 Institute of Biomedical and Clinical Science, University of Exeter Medical School, UK; 2 Department of Paediatric Endocrinology, Royal Manchester Children’s Hospital, UK; 3 Royal Devon and Exeter Foundation Hospital, Exeter, UK; 4 National Institute for Health Research Exeter Clinical Research Facility, University of Exeter Medical School, UK

## Abstract

**Objective:**

Mutations in the KATP channel genes, *ABCC8* and *KCNJ11*, are the most common cause of congenital hyperinsulinism. The diagnosis of KATP-hyperinsulinism is important for the clinical management of the condition. We aimed to determine the clinical features that help to identify KATP-hyperinsulinism at diagnosis.

**Design:**

We studied 761 individuals with KATP-hyperinsulinism and 862 probands with hyperinsulinism of unknown aetiology diagnosed before 6 months of age. All were referred as part of routine clinical care.

**Methods:**

We compared the clinical features of KATP-hyperinsulinism and unknown hyperinsulinism cases. We performed logistic regression and ROCanalysis to identify the features that predict KATP-hyperinsulinism.

**Results:**

Higher birth weight, diazoxide unresponsiveness and diagnosis in the first week of life were independently associated with KATP-hyperinsulinism (adjusted Odds Ratio 4.5 (95% CI, 3.4-5.9), 0.09 (0.06-0.13) and 3.3 (2.0- 5.0) respectively). Birth weight and diazoxide unresponsiveness were additive and highly discriminatory for identifying KATP-hyperinsulinism (ROC area under the curve for birth weight 0.80, diazoxide responsiveness 0.77, and together 0.88, 95% CI 0.85-0.90). 86% born large for gestation and 78% born appropriate for gestation who did not respond to diazoxide treatment had KATP-hyperinsulinism. In contrast, of those individuals born small for gestation, none who were diazoxide responsive and only 4% of those who were diazoxide unresponsive had KATP-hyperinsulinism.

**Conclusions:**

Individuals with hyperinsulinism born appropriate or large for gestation and unresponsive to diazoxide treatment are most likely to have an *ABCC8* or *KCNJ11* mutation. These patients should be prioritised for genetic testing for KATP channel genes.

Congenital hyperinsulinism (CHI) is a potentially life-threatening disorder characterised by inappropriately high levels of insulin at the time of hypoglycaemia. The incidence of CHI is estimated at 1 in 28,000 to 1 in 50,000 live births in European populations but rises to approximately 1 in 2,500 in countries with high rates of consanguineous unions ^1–3^.

Mutations in approximately 15 genes have been reported to cause isolated CHI or a multi-system syndromic disease where CHI is a rare feature ^4^. Screening these genes identifies a mutation in 36-69% of cases ^5, 6^. Inactivating mutations in the *ABCC8* and *KCNJ11* genes, encoding the SUR1 and Kir6.2 subunits of the pancreatic ATP-sensitive potassium (KATP) channel are responsible for 80-84% of confirmed monogenic cases of CHI ^5,6^.

Rapid screening of the KATP channel genes is critical for informing surgical and medical management of CHI ^7^. Identifying a dominantly-acting or bi-allelic *ABCC8* or *KCNJ11* mutation confirms a diagnosis of diffuse pancreatic disease which is preferentially managed with medical treatment whereas a paternally-inherited recessively-acting *ABCC8* or *KCNJ11* mutation suggest a focal pancreatic lesion which can be cured by lesionectomy ^8^.

In this study, we undertook genetic testing of the KATP channel genes in a large cohort of children with CHI presenting before the age of 6 months with the aim to assess whether clinical features at presentation could predict which individuals were most likely to have monogenic CHI due to a KATP channel mutation.

## Methods

### Study population and genetic analysis

We studied 761 probands with a pathogenic or likely pathogenic mutation in *ABCC8* (N=665) or *KCNJ11* (N=96) identified at the Exeter Genomics Laboratory between 2002 and 2018. We also reviewed 862 patients with CHI of unknown genetic aetiology who were referred to our laboratory within the same period. In all of these patients, mutations in the *ABCC8* and *KCNJ11* genes had been excluded by Sanger sequencing or gene panel testing.

Analysis of the coding regions and intron-exon boundaries of *ABCC8* and *KCNJ11* was performed using previously described methods ^9, 10^. When available, parental samples were tested to confirm the inheritance of *ABCC8*/*KCNJ11* variants identified in the proband. Pathogenicity of variants was assessed according to ACMG guidelines ^11^.

Clinical information was provided at referral for genetic testing using a standardised request form and included sex, ethnicity, birth weight, gestational age at birth, age at diagnosis of CHI, biochemical measurements at diagnosis (including insulin and glucose), current treatment, response to diazoxide treatment if attempted and the presence of additional features. Although a precise, consensus definition of diazoxide responsiveness is lacking, diazoxide unresponsiveness in this study can be broadly defined as persistent hypoglycaemia despite treatment with maximal dose diazoxide indicating the need for additional therapies to achieve euglycaemia. Consanguinity was defined as the parents of the proband being related as second cousins or closer or if the proband was referred from a country with high reported rates of consanguinity ^12^.

Informed consent was obtained from the parents or guardians of all probands. This study was approved by the North Wales Research Ethics Committee (517/WA/0327).

### Statistical analysis

Birth weight Z score and corrected birth weight for sex and gestation were generated using WHO standards accessed through the Zanthro package in Stata ^13^. Small for gestational age (SGA) was defined as a birth weight lower than the 10^th^ centile, and large for gestational age (LGA) was defined as a birth weight greater than the 90^th^ centile. Age at diagnosis followed a skewed distribution, so was analysed categorically between those diagnosed in the first week of life and those diagnosed after the first week. As insulin (pmol/l) level followed a right skewed distribution, values were log transformed for the statistical analysis.

Individuals with an *ABCC8* or *KCNJ11* mutation were combined (referred to as KATP channel mutations hereafter) as no difference in clinical features was observed between the two genetic subgroups (Supplementary Table 1). Statistical analyses were performed to determine differences in the clinical features of patients with an unknown aetiology compared to patients with a KATP channel mutation. Two-tailed P values were calculated to determine statistical significance using Pearson’s chi-squared test for categorical variables, and Student’s T test for continuous variables.

Univariate and multivariable logistic regression were used to assess which clinical features were independently predictive of having a KATP channel mutation. The area under the curve (AUC) of the receiver operator characteristic (ROC) was used to assess the discriminatory ability of the clinical features to identify individuals with a KATP channel mutation from those without a mutation. Stata/SE 16.0 (Stata Corp, College Station, TX, USA) was used to perform statistical analyses.

## Results

### Seven of the clinical features studied were different between individuals with KATP CHI and those with CHI of unknown aetiology

Individuals with KATP channel mutations (n=761) had higher birthweights (mean 4333g vs 3512g, *P*=6 x 10^-94^, mean difference 821g, 95% CI 748g-894g), were more likely to be diagnosed in the first week of life (85% vs 72%, *P*=1 x 10^-9^), had a higher insulin level at diagnosis (mean 162.2pmol/l vs 115.4pmol/l, *P*=1 x 10^-8^) and were less likely to respond to diazoxide, the mainstay treatment for CHI (32% vs 88%, *P*=2 x 10^-84^) compared to individuals with CHI of unknown genetic aetiology (n=862) (Table 1). They were more likely to be female (46% vs 36%, *P*=5 x 10^-5^), have consanguineous parents (52% vs 34%, *P*=2 x 10^-12^) and were less likely to be Caucasian (30% vs 52%, *P*=3 x 10^-18^) compared to CHI of unknown genetic aetiology. Glucose levels at presentation and the number of individuals with extra-pancreatic features at referral were similar between the two groups.

### Birth weight and diazoxide unresponsiveness are the most discriminative, independent, and additive for identifying children with KATP CHI

**Figure 1:**
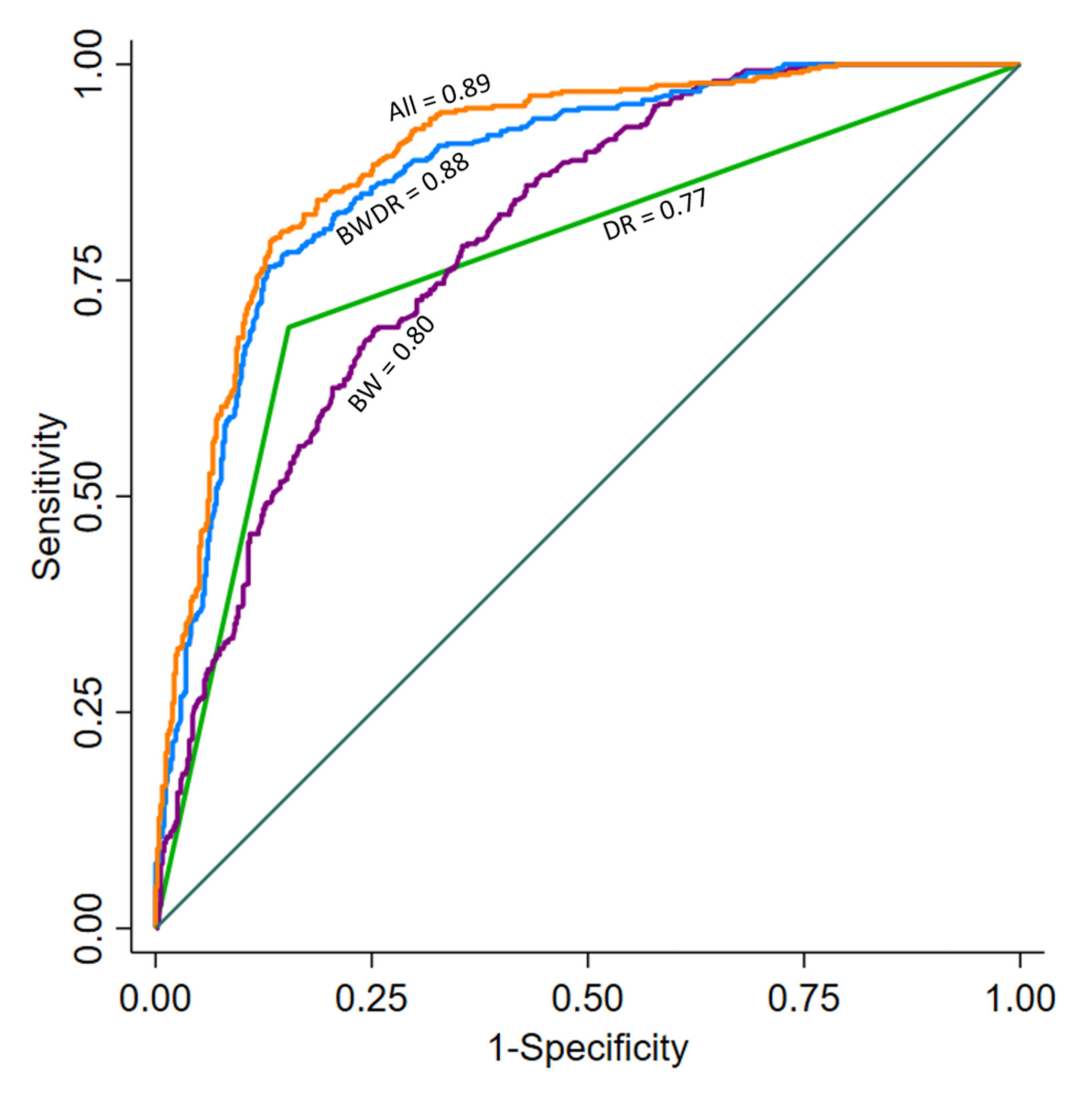
Receiver operating curves analysis showing the discriminating ability of clinical features to identify individuals with KATP CHI from those with unknown aetiology. DR denotes diazoxide responsiveness. BW denotes birth weight. All includes birth weight, diazoxide responsiveness, age at diagnosis, sex, insulin level at diagnosis, ethnicity, consanguinity.

To identify the independent features that can help in discriminating KATP CHI from unknown cases, we performed a multivariable logistic regression analysis. We showed that higher birth weight, diazoxide unresponsiveness, and a diagnosis within the first 7 days of life were independent predictors of KATP CHI after adjustment for all variables (Table 2). We next performed ROC AUC analysis to assess the discriminatory ability of these variables. The ROC AUC for birth weight was 0.80 (95% CI 0.77-0.82), 0.77 for diazoxide responsiveness (95% CI 0.74-0.80) and 0.59 for age at diagnosis (95% CI 0.57-0.62). Combining birth weight with diazoxide unresponsiveness increased the ROC AUC to 0.88 (95% CI 0.85-0.90). The addition of other factors (age at diagnosis, sex, ethnicity, consanguinity, insulin level) only marginally increased ROC AUC (0.89, 95% CI 0.87-0.91) over the combination of birth weight and diazoxide unresponsiveness (Table 2, Figure 1). To establish if our results were consistent across institutions that may have different standards in measuring these clinical features, we performed a sensitivity analysis with the two institutions that referred the most patients, along with the two countries with the most referred patients. We observed similar results in this analysis, suggesting the presence of homogeneity in clinical features despite different patient populations and clinical practice (Supplementary Table 2).

### Individuals born appropriate or large for gestation who did not respond to diazoxide had the highest likelihood of KATP CHI

**Figure 2:**
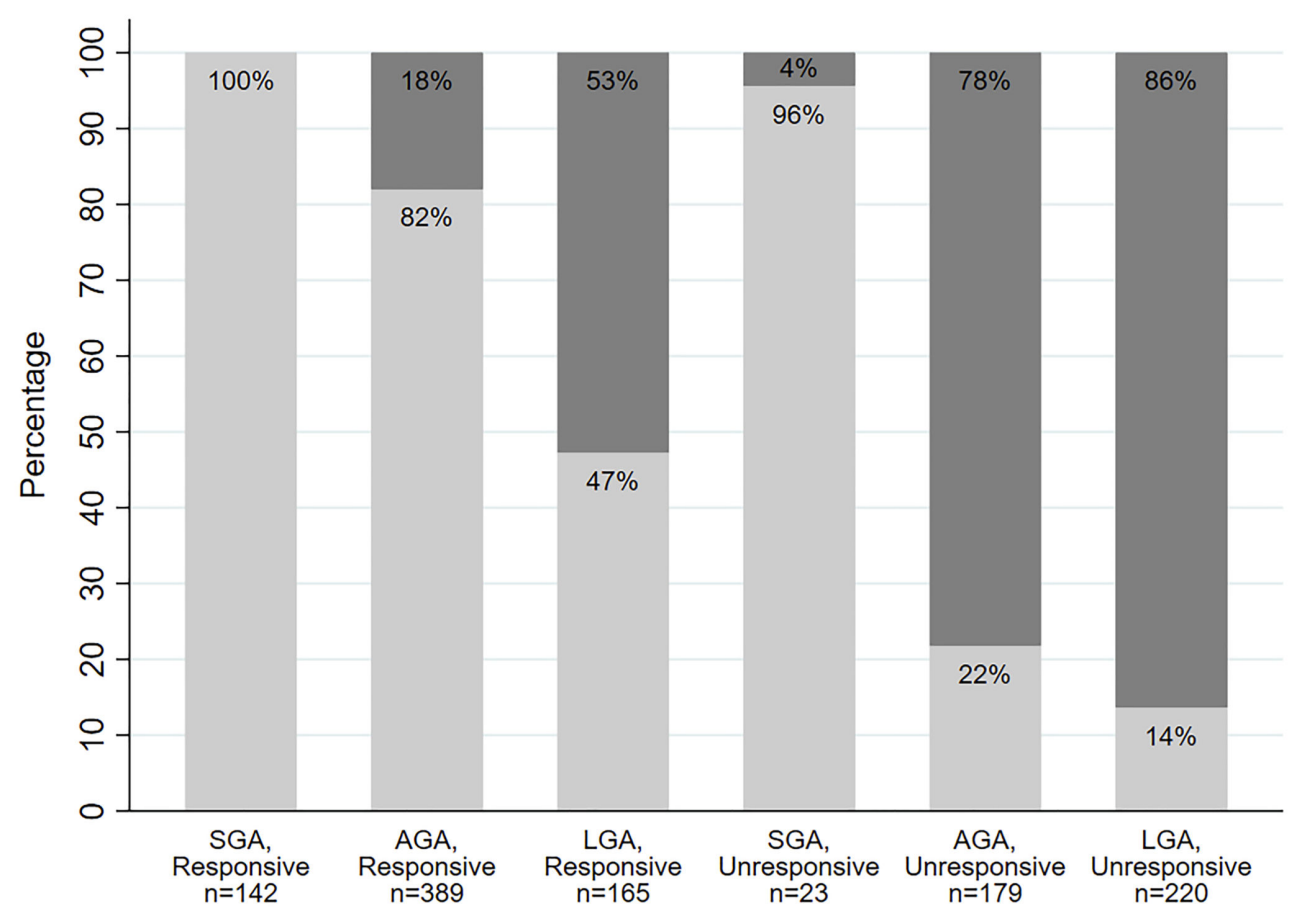
Proportion of KATP Congenital Hyperinsulinism by diazoxide responsiveness and birth weight categories. Light grey bars represent the percentage number of individuals without a KATP channel mutation, dark grey bars represent the percentage number of cases with a KATP channel mutation. SGA = small for gestational age, AGA = appropriate for gestational age, LGA = large for gestational age.

Of the 220 individuals born large for gestational age who were unresponsive to diazoxide, 86% (95% CI 81%-91%) had KATP CHI, whilst no mutations were detected in those born small for gestational age who responded to diazoxide (Figure 2). Of the 179 individuals born appropriate for gestational age who were unresponsive to diazoxide, 78% (95% CI 71%-84%) had KATP CHI, whilst of the 389 individuals born appropriate for gestational age who were responsive to diazoxide, only 18% (95% CI 14%-22%) had KATP CHI.

## Discussion

In this large study, we showed that simple clinical features such as birth weight and diazoxide responsiveness were independent and highly predictive for identifying individuals with KATP CHI from those with CHI of unknown aetiology.

We show that patients with KATP CHI were ~830 g heavier compared to CHI due to unknown cause. Higher birth weight has been reported previously in patients with KATP CHI ^14, 15^ and with persistent CHI ^16^. However, previous studies generally did not include control cases without KATP mutations, were smaller in size, and were limited to single centres impacting on the ability to statistically assess the importance of birth weight for identifying KATP CHI. We also show that birthweight and other clinical features were comparable between *ABCC8* and *KCNJ11* CHI thus this finding is applicable to both KATP HI subtypes (Supplementary Table 1). The higher birth weight in the KATP CHI patients is in keeping with the onset of hyperinsulinism with KATP CHI *in utero*, as insulin acts as a growth factor in pregnancy ^17^. This is supported by the observation that most of the children with CHI are diagnosed in the first week of life.

Diazoxide is the most common medical treatment for CHI which suppresses insulin secretion by binding to and opening the KATP channel ^18^. 78% of patients in our cohort who were unresponsive to diazoxide treatment had a KATP channel mutation, in keeping with the mutations leading an absence of channels or channels with disrupted function. Previous studies reported similar levels of diazoxide-unresponsiveness in patients with KATP CHI (5,6). Therefore, our finding is not unexpected, but due to large control cases, we were able to quantify the importance of lack of diazoxide response in identifying KATP CHI. Additionally, there was no difference in diazoxide responsiveness between patients with mutations in *ABCC8* and *KCNJ11* thus our findings are applicable to both genetic subtypes (Supplementary Table 1). Given that diazoxide is not universally available it will be important to perform further studies to assess whether responsiveness to other treatments (including somatostatin analogues) can help to predict KATP channel hyperinsulinism.

Our study has important clinical and research implications. Using the largest cohort of CHI cases referred from routine clinical practise, we robustly show that birth weight and response to diazoxide treatment can be used to help guide genetic testing in patients with CHI. Our cohort also included patients from multiple centres, of different ethnicities, and with different modes of inheritance for mutations in the KATP channel genes suggesting that our findings are applicable to patients worldwide. More than 80% of cases born LGA or AGA who were unresponsive to diazoxide had KATP channel mutations and therefore every effort should be made to prioritise genetic testing in these individuals as finding a KATP mutation can guide clinical management (7). Current Pediatric Endocrine Society guidelines on hypoglycaemia lack recommendation on criteria that can be used to prioritise patients for genetic testing for CHI (19). We believe our study provides important evidence in this regard which is especially relevant for resource-poor countries where access to genetic testing is limited ^19^. Furthermore, the findings from this study will help to prioritise patients for genetic discovery studies for patients with an unknown aetiology. Patients born large for gestational age who do not respond to diazoxide have an 86% chance of harbouring a mutation in a KATP channel gene (Figure 1). Therefore, patients in this category without a monogenic cause are good candidates to screen for pancreas-specific mosaic mutations and regulatory mutations affecting the KATP channel genes ^20^.

We acknowledge that our study has some limitations. We used clinician reported assessment of diazoxide responsiveness thus it is likely to vary by referring clinician. However, the rate of diazoxide unresponsiveness in KATP CHI is similar to studies that used a single definition for this feature ^5, 6^. Furthermore, the lack of consistent definition will have reduced the discriminatory ability rather than inflated the estimate suggesting that a robust definition will provide improved or similar results to our study. Our study did not include comprehensive data on whether hyperinsulinism was transient or persistent in each patient as we wanted to identify features at diagnosis which could help predict a KATP channel mutation. However, enrichment of male and SGA patients in the unknown aetiology group suggests the presence of transient HI as reported by a recent study ^21^. We used clinician reporting and country of referral to infer consanguinity. Although this approach has been used previously ^22^, we recognise that this has likely increased the proportion of consanguinity reported in our cohort. Despite this, consanguinity was significantly enriched in patients with KATP hyperinsulinism. This suggests that a more accurate definition will further increase its utility in identifying KATP hyperinsulinism. Finally, our study finding is not applicable to cases of non-KATP monogenic CHI. We chose to focus on KATP CHI as they are responsible for more than 80% monogenic CHI and finding these mutations is of great clinical importance.

In conclusion, this study shows that birth weight, diazoxide responsiveness and presenting with CHI in the first week of life are independent and highly predictive for KATP channel mutations in patients with congenital hyperinsulinism. Patients born normal or large for gestational age who are unresponsive to diazoxide are most likely to have KATP channel mutations and should be referred for genetic testing. Our study provides robust results that will help to shape future guidelines on genetic testing for CHI and is applicable to patients worldwide.

## Supplementary Material

Supplementary Figure 1

Supplementary Figure 2

## Figures and Tables

**Table 1:** 

Characteristics	CHI with confirmed KATP channel mutations	CHI of unknown aetiology	P value
**N**	761	862	-
**Age at diagnosis**	-	-	1 x 10^-9^
**≤7 days**	644 (85%)	622 (72%)	-
**>7 days**	117 (15%)	240 (28%)	-
**Female sex**	352 (46%)	313 (36%)	5x 10^-5^
**Corrected birth weight (g) [n]**	4333 (718) [746]	3512(762)[843]	6 x 10^-94^
**Birth weight Z score [n]**	1.64 (1.50) [746]	-0.13 (1.66) [843]	1 x 10^-95^
**Birth weight categories**	-	-	8 x 10^-93^
**LGA**	415 (56%) [746]	151 (18%) [843]	-
**AGA**	329 (44%) [746]	478 (57%) [843]	-
**SGA**	2 (0.3%) [746]	214 (25%) [843]	-
**Additional features**	102 (13%)	152 (18%)	0.02*
**White ethnicity [n]**	224 (30%) [736]	438 (52%) [841]	3 x 10^-18^
**Consanguineous parents**	394 (52%)	296 (34%)	2 x 10^-12^
**Glucose (mmol/L) [n]**	1.6 (0.7) [653]	1.7 (0.8) [740]	0.02*
**Insulin (pmol/L) [n]**	162.2 (3.0) [666]	115.4 (2.9) [715]	1 x 10^-8^
**Diazoxide responsive [n]**	160 (32%) [495]	521 (88%) [591]	2 x 10^-84^

**Table 2:** 

Characteristics	Unadjusted Odds ratio (95% CI)	P value	Adjusted Odds ratio (95% CI)	P value
**Diagnosed in first** **week of life**	2 (1.6 -2.5)	3 x 10^-10^	3.3 (2.0- 5.0)	2 x 10^-6^
**Female sex**	1.5 (1.2 – 1.8)	5 x 10^-5^	0.9 (0.6 – 1.3)	0.6
**Corrected birth** **weight**	4.4 (3.7 – 5.2)	3 x 10^-68^	4.5 (3.4 – 5.9)	2 x 10^-26^
**Caucasian ethnicity**	2.4 (2.0 – 3.0)	9 x 10^-18^	1.5 (0.9 – 2.6)	0.1
**Consanguinity**	2.0 (1.6 – 2.4)	7 x 10^-12^	1.9 (1.1 – 3.3)	0.02
**Insulin at diagnosis**	1.9 (1.5 – 2.4)	9 x 10^-9^	0.7 (0.5 – 1.0)	0.08
**Diazoxide** **responsive**	0.08 (0.06 – 0.11)	2 x 10^-65^	0.09 (0.06 – 0.13)	2 x 10^-36^
