# Supplementary material for: Birth weight and diazoxide unresponsiveness strongly predict the likelihood of congenital hyperinsulinism due to a mutation in ABCC8 or KCNJ11
Source: Eur J Endocrinol. Author manuscript; Available in PMC 2021 Nov 11. (PMC7611977; doi:10.1530/EJE-21-0476)
Supplement: Supplementary Figure 1 [file EMS137877-supplement-Supplementary_Figure_1.docx]

Supplementary Table 1 – Characteristics of individuals with congenital hyperinsulinism caused by mutations in ABCC8 and KCNJ11. Categorical data are shown as n (%) whereas continuous data are shown as mean (SD). *P values are above the Bonferroni corrected threshold for multiple comparison (0.05/8 = 0.006). Number of patients [n] is shown In square brackets where it differs from the total number in the cohort.

| **Characteristics** | **CHI with confirmed *ABCC8* mutation** | **CHI with confirmed *KCNJ11* mutation** | **P value** |
| --- | --- | --- | --- |
| **N** | 665 | 96 | - |
| **Age at diagnosis** | - | - | 0.88 |
| **≤7 days** | 562 (85%) | 82 (85%) | - |
| **>7 days** | 103 (15%) | 14 (15%) | - |
| **Female sex** | 313 (47%) | 39 (41%) | 0.27 |
| **Corrected birth weight (g) [n]** | 4331 (706) [650] | 4341 (797) | 0.90 |
| **Birth weight Z score [n]** | 1.64 (1.47) [650] | 1.65 (1.66) | 0.93 |
| **Birth weight categories** | - | - | 0.19 |
| **LGA** | 365 (56%) [650] | 50 (52%) | - |
| **AGA** | 284 (44%) [650] | 45 (47%) | - |
| **SGA** | 1 (0.2%) [650] | 1 (1%) | - |
| **Additional features** | 84 (13%) | 18 (19%) | 0.11 |
| **White ethnicity [n]** | 195 (30%) [642] | 29 (31%) [94] | 0.91 |
| **Consanguineous parents** | 343 (52%) | 51 (53%) | 0.83 |
| **Glucose (mmol/L) [n]** | 1.6 (0.7) [577] | 1.6 (0.6) [76] | 0.90 |
| **Insulin (pmol/L) [n]** | 161.2 (3.0) [588] | 170.0 (2.9) [78] | 0.69 |
| **Diazoxide responsive [n]** | 133 (31%) [423] | 27 (38%) [72] | 0.34 |
