# Supplementary material for: Birth weight and diazoxide unresponsiveness strongly predict the likelihood of congenital hyperinsulinism due to a mutation in ABCC8 or KCNJ11
Source: Eur J Endocrinol. Author manuscript; Available in PMC 2021 Nov 11. (PMC7611977; doi:10.1530/EJE-21-0476)
Supplement: Supplementary Figure 2 [file EMS137877-supplement-Supplementary_Figure_2.docx]

**Supplementary Table 2 - Sensitivity analysis showing the utility of clinical features to discriminate between KATP CHI and CHI of an unknown aetiology in the two countries and institutes with the most referrals. ROC AUC = Receiver Operating Characteristic Area Under the Curve**

|  | **No with KATP HI/total HI** | **ROC AUC for corrected birth weight alone (95% CI)** | **ROC AUC for diazoxide responsiveness alone (95% CI)** | **ROC AUC for corrected birth weight and diazoxide responsiveness (95% CI)** | **ROC AUC for corrected birth weight, diazoxide responsiveness, sex, insulin, ethnicity, consanguinity (95% CI)** |
| --- | --- | --- | --- | --- | --- |
| **Whole cohort** | 761/1685 | 0.80 (0.77 – 0.82) | 0.77 (0.74 – 0.80) | 0.88 (0.85 – 0.90) | 0.89 (0.87 – 0.91) |
| **Patients from the UK** | 173/531 | 0.85 (0.80 – 0.89) | 0.84 (0.79 – 0.88) | 0.94 (0.91 – 0.96) | 0.93 (0.91 – 0.96) |
| **Patients from Turkey** | 102/208 | 0.86 (0.80 – 0.92) | 0.75 (0.68 – 0.82) | 0.89 (0.84 – 0.95) | 0.90 (0.85 – 0.96) |
| **Patients referred from GOSH** | 116/283 | 0.81 (0.74 – 0.87) | 0.84 (0.79 – 0.90) | 0.91 (0.87 – 0.96) | 0.93 (0.88 – 0.97) |
| **Patients referred from Manchester** | 35/90 | 0.89 (0.81 – 0.98) | 0.80 (0.69 – 0.91) | 0.94 (0.87 – 1.00) | 0.93 (0.86 – 0.99) |
